# Differential effects of familiarity and emotional expression of musical cues on autobiographical memory properties

**DOI:** 10.1177/17470218221129793

**Published:** 2022-10-27

**Authors:** Kelly Jakubowski, Emma Francini

**Affiliations:** 1 Department of Music, Durham University, Durham, UK; 2 Department of Psychology, Durham University, Durham, UK

**Keywords:** Autobiographical memory, music-evoked autobiographical memory, emotion, familiarity, retrieval cues

## Abstract

Features of visual cues, such as their familiarity and emotionality, influence the quantity and qualities of the autobiographical memories they evoke. Despite increasing use in autobiographical memory research, comparatively little is known about how such features of musical cues influence memory properties. In a repeated-measures design, we presented 24 musical cues selected to vary on their familiarity (high/low), emotional valence (positive/negative), and emotional arousal (high/low) to 100 young adults, who recorded details of any autobiographical memories that were evoked. Familiarity of the music primarily impacted memory accessibility, with high-familiarity music evoking more memories that were retrieved more quickly. More familiar music also elicited more positive and arousing memories; however, these differences were found to be attributed to greater liking of the high-familiarity music. The emotional expression of the music impacted the emotionality and evaluation of the memories, with negative valence/low-arousal (e.g., “sad”) music evoking the most negative memories, high-arousal and positively valenced music evoking more arousing memories, and low-arousal music evoking memories rated as more important. These results provide important insights for developing effective paradigms for triggering (particular types of) autobiographical memories via music and highlight the need to critically consider potential differences in cue familiarity and emotionality in studies comparing musical with non-musical cues. Future research should extend this approach to other cue types (e.g., visual, olfactory, other auditory cues), to probe how familiarity and emotional qualities of cues conjunctively or interactively constrain autobiographical memory recall across different domains.

It is well established that differences in features of retrieval cues can influence the accessibility and phenomenological qualities of autobiographical memories. For example, studies of visual cues have shown that more familiar cues elicit more frequent autobiographical memories (Robin et al., 2019; Robin & Moscovitch, 2014, 2017) and the emotional tone of cues can impact the content and emotionality of the associated memories (Simpson & Sheldon, 2020). The modality (e.g., visual, auditory, olfactory) via which retrieval cues are presented also affects both the quantity and phenomenological qualities of autobiographical memories (Congleton et al., 2021; Herz, 2004; Herz & Schooler, 2002). Studies using the cue-word method (Crovitz & Schiffman, 1974) have shown that the imageability and concreteness of the word cues positively impact the quantity, retrieval speed, and specificity of autobiographical memories (Robinson, 1976; Rubin & Schulkind, 1997; Uzer, 2016; Uzer et al., 2012; Williams et al., 1999).

Another retrieval cue that is increasingly used in both behavioural (Janata et al., 2007; Sheldon & Donahue, 2017; Zator & Katz, 2017) and neuroscientific (Ford et al., 2011, 2016; Janata, 2009) research on autobiographical memory is music (Belfi & Jakubowski, 2021). Music is a particularly useful means for accessing autobiographical memories because it is a stimulus to which most people in the Western world are exposed on a daily basis (Juslin & Laukka, 2004; Sloboda et al., 2001), many important events in our lives are accompanied by music (Merriam, 1964), and particular pieces of music are often associated with particular life periods (Cady et al., 2008). Music has been shown to be an effective cue for sampling over a wide range of autobiographical memories; for instance, music cues evoke memories that vary naturally in their level of specificity (Ford et al., 2011), and the diverse emotions expressed by music can elicit a similarly diverse range of emotional memories (Sheldon & Donahue, 2017; Sheldon et al., 2020). Music also plays a key role in identity formation (Tarrant et al., 2002), shows a reminiscence bump similar to other common autobiographical memory cuing methods (Jakubowski et al., 2020; Platz et al., 2015; Rathbone et al., 2017), and is a particularly useful medium for studying cultural transmission across generations (Krumhansl & Zupnick, 2013). In addition, music has been shown to be an effective cue for positive, self-defining autobiographical memories in people with early-stage Alzheimer’s disease (El Haj et al., 2012, 2015).

Despite accumulating evidence on the overall nature of music-evoked autobiographical memories (MEAMs) (Jakubowski & Ghosh, 2021; Janata et al., 2007), less is known about how features of specific pieces of music impact the memories they evoke. This is in stark contrast to autobiographical memory cuing methods with a longer history of use, such as the cue-word method, where the influence of various features of the cues (e.g., imageability, concreteness, object vs affect words) on memory properties have been extensively explored (Robinson, 1976; Rubin & Schulkind, 1997; Uzer et al., 2012). Understanding how features of musical stimuli impact memory properties is important for designing effective paradigms for triggering MEAMs and may also inform the development of clinical interventions aiming to use music as a tool for accessing certain types of memories (e.g., positive or self-defining memories). In addition, such work will facilitate more controlled future comparisons between music and other retrieval cues. Specifically, although a range of previous studies have compared music to other cues (e.g., images, words, sounds), these studies have generally not accounted for potential differences between the musical and non-musical cues they used in terms of key features such as modality, familiarity/prior exposure, liking, and emotionality^1^ (Belfi et al., 2016, 2020; Jakubowski et al., 2021; Zator & Katz, 2017). In this experiment, we began to examine this question of how features of particular musical stimuli impact memory qualities by manipulating and testing the effects of familiarity and emotional expression of musical cues on the quantity and properties of the autobiographical memories they evoked.

## Familiarity of cues

Repeated exposure to a retrieval cue can strengthen the association between the cue and an autobiographical memory via reactivation and rehearsal. For instance, research has shown that autobiographical memories are recalled more frequently, faster, in more detail, and more vividly when cued by more familiar spatial contexts (Robin et al., 2019; Robin & Moscovitch, 2014, 2017) However, the cue overload principle also predicts that greater familiarity with a cue will lead to retrieval of less specific memories, given that the cue is likely to have been previously associated with a variety of different autobiographical contexts (Watkins & Watkins, 1975, see also Robin et al., 2019).

Several previous studies have found positive correlations between familiarity and the autobiographical salience of pieces of music (Jakubowski et al., 2020; Janata et al., 2007; Krumhansl & Zupnick, 2013; Schulkind et al., 1999). That is, songs that were better recognised or rated as more familiar were more likely to be associated with autobiographical memories. Research comparing older adults with and without Alzheimer’s disease has revealed that, in comparison to experimenter-selected music, self-selected music (which is presumably more familiar, although this was not explicitly measured) elicited memory descriptions comprising more positive and fewer negative emotion words in both groups (El Haj et al., 2012). Similarly, Ford et al. (2016) found that pop songs rated as more familiar elicited more positive memories, and this relationship was stronger in older than younger adults. More familiar music was also associated with memories rated as more specific, but only in young adults. This may be related to a general shift observed in older adults to recall less specific but more positive autobiographical memories (Carstensen et al., 1999; Levine et al., 2002; Mather & Carstensen, 2005).

In sum, more familiar music appears to be more likely to evoke autobiographical memories, which may be more positive and specific in nature. A limitation of previous research is that the relationship between cue familiarity and MEAMs has only been investigated through correlational designs, and thus causal inferences cannot be definitively made. In addition, the potential interactive effects of the familiarity and emotional expression of musical cues on MEAMs have not been investigated, as experiments on the impact of the emotional expression of musical cues on MEAMs have used solely unfamiliar music to date (Jakubowski & Eerola, 2022; Schulkind & Woldorf, 2005; Sheldon & Donahue, 2017; Sheldon et al., 2020).

## Emotional expression of cues

Previous research has demonstrated that it is easier to access autobiographical memories of a similar emotional tone to one’s current mood (Blaney, 1986; Bower, 1981; Singer & Salovey, 1988). More recent research has found that mood state manipulations have less reliable effects on memory content in comparison with the emotional tone of a specific retrieval cue (Simpson & Sheldon, 2020). Four studies have further investigated effects of the emotional tone of retrieval cues on autobiographical memory using unfamiliar musical cues. Three of these found that music expressing positive valence evoked more positive autobiographical memories than negatively valenced music (Schulkind & Woldorf, 2005; Sheldon & Donahue, 2017; Sheldon et al., 2020), although one study found that music cued relatively positive memories regardless of its emotional valence (Jakubowski & Eerola, 2022). This effect was specific to music, as Jakubowski and Eerola (2022) also found that environmental sounds and single words both showed a valence congruence effect, with more positively valenced cues evoking more positive memories. They suggested that this finding may be related to the fact that engaging with negative (e.g., “sad” and “angry”) music often elicits positive emotional responses due to the lack of “real-life” consequences imposed by negative aesthetic stimuli such as music (see also the studies by Eerola et al., 2018; Thompson et al., 2019). Two of the aforementioned studies also revealed that more arousing music evoked autobiographical memories rated as more energetic, suggesting an arousal congruence effect, although this effect was not found when arousal was instead measured as “emotional intensity” (Jakubowski & Eerola, 2022; Sheldon & Donahue, 2017).

Beyond emotional congruence effects, several other notable findings were revealed in these studies. To summarise briefly, Schulkind and Woldorf (2005) found that positively valenced music also evoked more arousing memories, and a significant interaction effect between cue valence and cue arousal on memory valence indicated that negative valence/low-arousal stimuli (e.g., “sad music”) elicited the least positive memories. In addition, Sheldon and Donahue (2017) found that positively valenced cues elicited more social and energetic memories than negative cues, and high-arousal cues elicited more social, less vivid, and less unique memories than low-arousal cues. Finally, both positively valenced cues and high-arousal cues led to faster memory retrieval times in three studies (Schulkind & Woldorf, 2005; Sheldon & Donahue, 2017; Sheldon et al., 2020), with Sheldon and Donahue (2017) also reporting a significant interaction effect, in which “happy” (positive valence/high-arousal) cues evoked memories significantly faster than the other valence/arousal categories.

In sum, previous studies have provided some (albeit mixed) evidence for emotional congruence effects between musical cues and associated autobiographical memories, and have also demonstrated that the emotional expression of musical cues can impact other features of the memories, such as retrieval speed, vividness, and ratings of memory content. As mentioned above, all studies on the effects of music’s emotional expression on MEAMs to date have used unfamiliar music. As such, it is unclear whether the effects reported above generalise to familiar music, or whether the familiarity and emotional expression of musical cues show interactive effects on MEAM properties. For example, it could be that the emotional expression of a musical cue guides a listener towards retrieval of an emotionally congruent memory only in the case of unfamiliar music, whereas familiar music that has already been coupled to previous life events may not exhibit such a congruency effect.

## The present study

We varied both the familiarity and emotional expression of musical stimuli to investigate their effects on the number and qualities of associated autobiographical memories. Specifically, we preselected musical cues with high and low familiarity (based on pilot studies) that fell into each of the four quadrants of the circumplex, two-dimensional model of emotion (Posner et al., 2005; Russell, 1980), in which emotions are conceptualised by their valence (variations in positivity/negativity) and arousal (variations in activation/deactivation). To maximise the familiarity differences between musical cues, we constrained our sample to young adults (aged 18–29 years) who were born in and currently living in the United Kingdom while acknowledging that future work will be needed to explore these research questions in other populations.

In line with previous research, we predicted that high-familiarity music would evoke more memories, and more positive memories, than music with low familiarity. We also predicted that the emotional expression of the musical cues would show some congruence with the emotions of the memories evoked. In addition to the number and emotions of the memories evoked, we measured memory retrieval times and took ratings of the vividness, uniqueness, and importance of each memory, to further explore the effects of the music’s familiarity and emotional expression on these key memory features. In addition, we investigated the interactive effects of the familiarity and emotional expression of the musical cues on MEAM properties, to test whether, for instance, the emotional expression of music has different effects on MEAMs depending on the familiarity level of the music. As such interactions have not been investigated before, we did not make a priori hypotheses in this regard, but explored these comprehensively to gain a fuller understanding of how these two key features of music direct autobiographical recall. The results of this experiment contribute new and necessary insights into how particular music can be used to access different episodes from across our personal histories.

## Method

### Design

In an online experiment, we tested the effects of familiarity (high/low), emotional valence (positive/negative), and emotional arousal (high/low) of musical cues on properties of associated autobiographical memories. The dependent variables were the number of memories evoked, retrieval times, and ratings of several key properties of the memories (emotional valence, emotional arousal, vividness, uniqueness, and importance).

### Participants

A power analysis was run in G*Power (Faul et al., 2007) using the effect size reported for a three-way interaction ( $\eta_{p}^{2}=.119$ ) in an experiment using a similar design to the present work (Sheldon & Donahue, 2017), indicating a sample size of 66 participants was required to achieve 80% power. We thereby recruited 101 adult participants. One participant was excluded due to technical difficulties in sound playback, leaving a final sample size of 100 (ages 18–29 years, *M* = 23.44, *SD* = 3.42, 71 females and 29 males). All participants were born in and currently living in the United Kingdom, and all spoke English as their native language. They were required to confirm that they had no previous history of any of the following: stroke, severe head injury, brain tumour/injury, any other neurological condition that may contribute to cognitive impairment, severe depression or anxiety, alcohol abuse or dependence, or recurrent substance abuse or dependence. In response to a self-report question, no participant reported any history of hearing loss. In terms of musical background, 82% of participants reported they were non-musicians while 18% identified as amateur musicians, and 73% reported two or fewer years of formal musical training (see online Supplementary Material A for all demographic and musicianship questions). All were panellists from Prolific (https://www.prolific.co) and were compensated (£4.38) for their participation.

### Materials/stimuli

#### Musical stimuli

As we aimed to study the influence of music (rather than the semantic connotations of lyrics) on autobiographical memory, we used stimuli comprising only instrumental sections of pieces of music, which occasionally—in the case of six stimuli (five high-familiarity stimuli, one low-familiarity stimulus)—contained nonsense syllables or other vocalisations (e.g., “la la la,” “ohhh,” shouts, whistles). Thus, for both the high- and low-familiarity musical stimuli, we selected pieces of music that had an instrumental-only section (or instrumental section with occasional nonsense vocalisations) of 10 s or more. All musical stimuli were edited to 30 s in duration by looping the selected section as necessary.^2^ A stimulus duration of 30 s was chosen based on previous research that used the same low-familiarity stimuli as autobiographical memory cues (Jakubowski & Eerola, 2022).

The high-familiarity musical stimuli were songs that had featured in the UK charts and comprised a mix of recent hits (e.g., “Thinking Out Loud” by Ed Sheeran) and older but still widely recognised “classics” (e.g., “Here Comes the Sun” by the Beatles). Stimuli were sourced by consulting UK Top 100 Singles Charts from the past 10 years, lists of the all-time top-selling singles in the United Kingdom^3^, and lists of well-known pop songs used in previous music psychology studies with UK participants (Frieler et al., 2013). From these sources, we compiled a large list of potential stimuli, which were then judged independently by the two authors in terms of their familiarity and whether they expressed positive/negative valence and high/low arousal. A total of 44 musical excerpts were then selected for piloting, with 11 songs per valence/arousal category.

To verify the perceived valence and arousal of the excerpts, as well as their familiarity, an online pilot study was conducted with 139 participants. The sampling criteria for this pilot study were the same as the main experiment. The mean age of this pilot sample was 23.19 years (*SD* = 3.75, range = 18–32; 76 females, 61 males, 2 others). All participants were born in and currently residing in the United Kingdom, and all spoke English as their native language. Most participants categorised themselves as non-musicians (75%), and 68% reported two or fewer years of formal musical training. Two participants reported some bilateral hearing loss, with one wearing hearing aids in both ears and the other taking no corrective measures. After providing informed consent and completing demographic questions, participants listened to 22 of the musical excerpts (distributed equally so that each excerpt was rated by half the sample). For each excerpt, they were asked to rate the extent to which it expressed a negative/positive emotion and a low/high level of energy (both on 9-point scales). They were also asked whether they had ever heard the piece of music before (with “yes,” “maybe,” and “no” as response options), and if they answered “yes” or “maybe” were asked to name the song and the performer. The results showed that arousal was clearly differentiated into high/low categories by the participants; the average arousal ratings by musical excerpt showed a pronounced bimodal distribution, with 20 excerpts exhibiting an average arousal rating of less than 5 and the other 24 excerpts being given an average arousal rating above 6. Average valence ratings by excerpts were negatively skewed and also correlated with arousal ratings (*r* = .77). To remove this skew, we scaled the average valence ratings onto a 1–9 scale for each arousal category (high/low) separately. For the main experiment, we selected three musical excerpts from each of the four valence/arousal quadrants^4^ that were rated as familiar (“yes” response to the familiarity question^5^) by at least 66.7% of pilot participants.

For the low-familiarity music, we utilised the same 12 stimuli used by Jakubowski and Eerola (2022) in Experiments 3 and 4 of their article. These stimuli were sourced from the MediaEval Database for Emotional Analysis in Music (DEAM), which comprises a set of royalty-free music of varying genres (Aljanaki et al., 2017). These pieces of music were rated as largely unfamiliar to participants in the initial curation of the corpus by Aljanaki et al. (2017), as well as by participants in the study by Jakubowski and Eerola (2022), who utilised a highly similar sample to this study (native English speakers with UK nationality, primarily young adults). The DEAM database also includes participant ratings of the perceived valence and arousal of each piece of music; we utilised three stimuli from each of the four valence/arousal quadrants.

The full list of stimuli used in the main experiment can be found in the online Supplementary Material B. For each stimulus, we list its familiarity category, emotional valence and arousal category, title, artist, genre, and (for the commercially released, high-familiarity music) year of release.

Finally, to further validate our stimulus selection, we ran an online study with 81 participants from the same demographic as our main experiment (all were native English speakers born in and currently residing in the United Kingdom, aged 18–29 years, *M* = 25.11, *SD* = 2.96, 58 females, 22 males, 1 other). Half (41) of the participants rated the 12 low-familiarity stimuli and the other 40 rated the high-familiarity stimuli on their perceived valence (negative/positive emotion) and arousal (low/high level of energy) on 5-point scales. The stimuli we had categorised as high arousal (*M* = 4.47, *SD* = 0.25) were rated significantly higher in arousal than the stimuli we categorised as low arousal (*M* = 2.22, *SD* = 0.61), *t*(15) = 11.90, *p* < .001. Similarly, the stimuli we categorised as positive valence (*M* = 3.89, *SD* = 0.56) were rated as significantly more positive than those in the negative valence category (*M* = 2.47, *SD* = 0.59), *t*(22) = –5.99, *p* < .001. Importantly, the high- and low-familiarity stimuli did not differ overall in ratings of perceived valence, *t*(22) = 1.29, *p* = .21, or perceived arousal, *t*(22) = –0.19, *p* = .85, indicating these were well matched overall.

#### Instructions and measures

The experiment was run online, implemented through Qualtrics. Participants were instructed to use each piece of music that they heard to help them think of an autobiographical memory. An autobiographical memory was defined to them as “A memory of an event that you were personally involved in, involving a specific place and time, that lasted no longer than one day.” They were also provided an example: “For example, the sound of a string quartet playing a piece of classical music might bring back a memory of your sister’s wedding that you attended last July in a park in London.” They were informed that each piece of music would begin playing automatically, and they should press a button reading “I have recalled a memory” as soon as an autobiographical memory came to mind. If no memory came to mind, they were asked to refrain from pressing the button and wait for the next piece of music to begin; the experiment advanced to the next trial automatically after 30 s if no memory was reported.

Ratings of each retrieved memory’s emotional valence, emotional arousal, vividness, uniqueness, and importance were made on 5-point Likert-type scales. We also asked participants to provide a short (one sentence) written description of each memory, report how old they were when the event originally occurred, and assess whether the piece of music that they had just heard was present during the event they had recalled. We calculated memory-retrieval time as the amount of time between the initial presentation of a musical stimulus and the time a participant clicked the button reading “I have recalled a memory.” Regardless of whether a memory was recalled, for each musical stimulus, participants also rated how familiar they were with the piece of music and how much they liked it on 5-point scales. If they gave a familiarity rating higher than “1 (*never heard it before*)” they were also asked to type the name of the piece of music. Participants’ musical backgrounds were assessed using one question from the Ollen Musical Sophistication Index on their level of musicianship (Ollen, 2006) and one question from the Goldsmiths Musical Sophistication Index on their amount of previous musical training (Müllensiefen et al., 2014). See online Supplementary Material A for wording of all questions and rating scales described above.

### Procedure

After providing informed consent, participants completed demographic questions (e.g., age, gender, see Supplementary Material A). Next, they completed a sound check, during which they were asked to adjust their device volume to a comfortable level; they were asked to keep the volume at this same level throughout the experiment. They then saw the instructions for the main experiment and completed one practice trial using an excerpt of music from the DEAM database that was not used as a stimulus in the main experiment. They subsequently completed the main experiment, in which they heard all 24 musical stimuli in a randomised order and were asked to report and provide ratings of any autobiographical memories that came to mind. Finally, the two questions on their musical background were completed. The median time taken to complete the experiment was 28.49 min.

### Analysis

Data were analysed in R. Our primary analyses focused on testing the main effects and interactions of familiarity, emotional valence, and emotional arousal of the musical cues on the accessibility, emotional content, and other key qualities of the autobiographical memories. We employed mixed-effects models with “participant” as a random effect in each model, given the repeated-measures nature of the data. A binomial mixed-effects model was fitted to predict whether a memory was generated or not (“retrieval success,” as a binary variable); all other dependent variables were investigated using linear mixed-effects models. As we investigated the effects of our predictor variables on seven different dependent variables, we adopted a conservative, Bonferroni-corrected significance level of .05/7 = .0071. All mixed-effect models were fitted using the “lme4” R package (Bates et al., 2015), and the overall statistical significance of each of the fixed effects/interactions was assessed with Wald χ^2^ tests via the analysis of variance (ANOVA) function in the “car” package (Fox & Weisberg, 2019). Estimated marginal means (EMMs) were computed using the “emmeans” package (Lenth, 2022), and Nagelkerke pseudo-*R*^2^ values for the models were computed using the “rcompanion” package (Mangiafico, 2022).

## Results

### Descriptive statistics, familiarity manipulation check, and age of memories

Data collected in this experiment can be accessed via the Open Science Framework (https://osf.io/3hucm/). In total, 1,085 autobiographical memories were evoked in the experiment, that is, 45% of the cues presented to participants evoked memories. On average, participants reported 11 memories (*SD* = 4, range = 1–24). All musical stimuli evoked some memories, although the number of memories cued by any one song varied between 15 and 78 memories.

The musical stimuli we had assigned to the high-familiarity category were rated significantly higher on the 5-point familiarity rating scale (*M* = 4.35, *SD* = 0.64, 95% confidence interval [95% CI] = [4.22, 4.48]) than those assigned to the low-familiarity category (*M* = 1.25, *SD* = 0.24, 95% CI = [1.20, 1.30]) in a paired-samples *t*-test, *t*(99) = 46.57, *p* < .001. In addition, on 71.31% of high-familiarity music trials, overall participants were able to recall the correct name of the song^6^, whereas they were unable to name the song title on any of the low-familiarity trials. On 78.70% of high-familiarity trials and 3.83% of low-familiarity trials in which a memory was recalled, participants reported that they thought that particular song was present during the original event (i.e., at encoding).

To further ensure the validity of our familiarity manipulation on a trial-level basis, we excluded from all subsequent analyses all trials in which a participant gave a stimulus assigned to the high-familiarity category a rating of “1 (*never heard it before*)” or “2” on the familiarity rating scale (*N* = 110 trials overall; 4.6% of the dataset). Analogously, we excluded all trials in which a participant gave a stimulus assigned to the low-familiarity category a rating of “4” or “5 (*have frequently heard it*)” on the familiarity rating scale (*N* = 11 trials overall; 0.5% of the dataset).

Participants recalled events from when they were aged 4 to 29 years, with a mean age at event of 16.78 years (*SD* = 5.28). These data showed a recency effect, with 9.05% of all memories reported being of events that had happened in the past year and 50.34% being of events from the past 5 years. Supplementary Figure 1 shows the distribution of (1) the participant’s age at event and (2) the age of the memory for all reported memories. To test whether the participant’s age at event varied systematically in relation to the cue features, we fitted a linear mixed-effects model with familiarity, emotional valence, and emotional arousal of the music cues as predictors of age at event and “participant” as a random effect. This analysis revealed a significant three-way interaction between familiarity, emotional valence, and emotional arousal of the cues, χ^2^(1) = 11.20, *p* < .001. In post hoc pairwise comparisons within Bonferroni correction, we found that the high-familiarity music that expressed negative valence and high arousal evoked older memories than all other cue categories (all *p*s < .004) except the low-familiarity music that expressed positive valence and high arousal (*p* > .99). All other pairwise comparisons were non-significant (*p*s > .29), indicating that, for the most part, the different cue categories evoked memories from a similar time period.

### Effects of familiarity and emotional expression of cues on autobiographical memory properties

**Figure 1.**
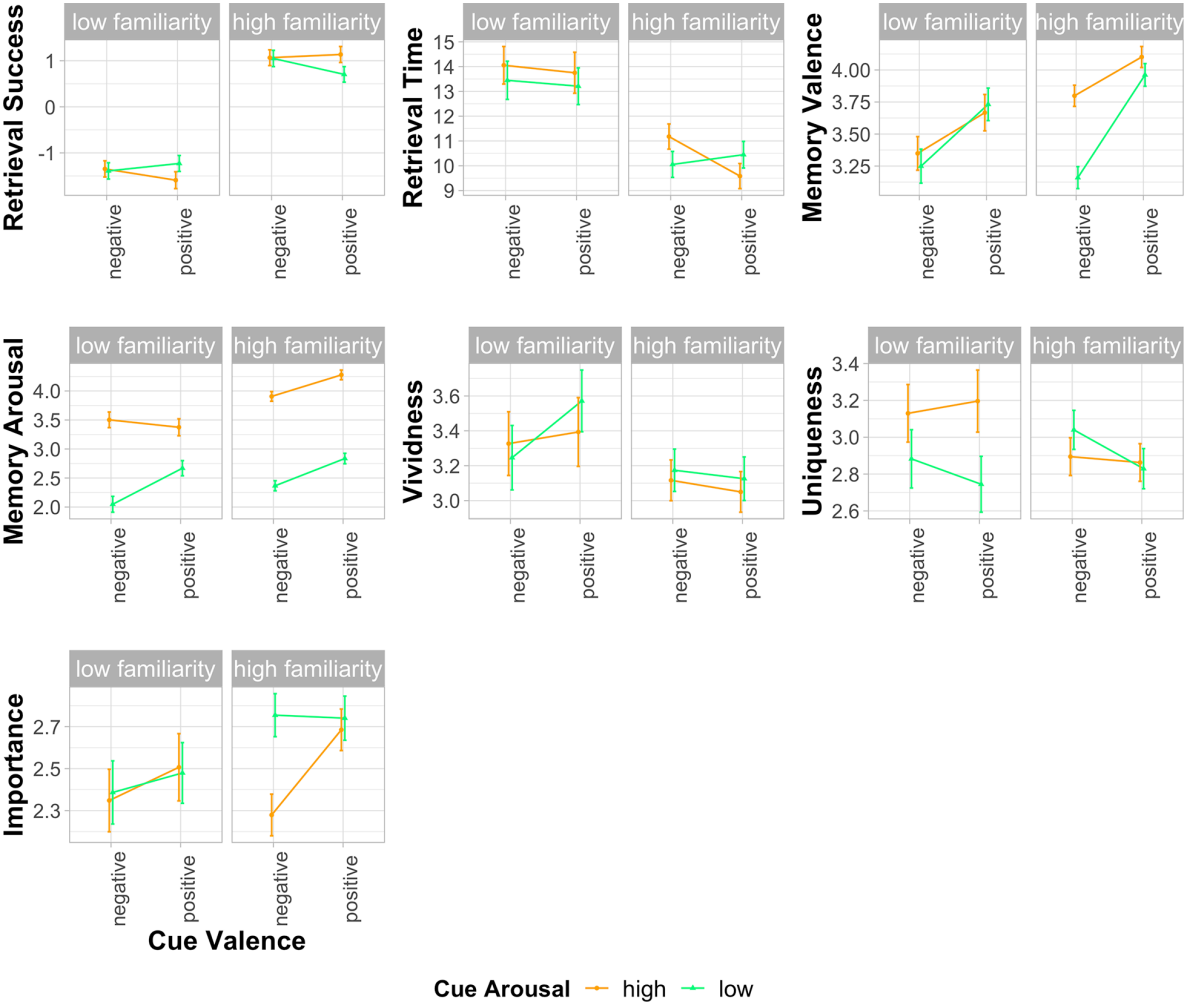
Estimated marginal means by cue familiarity, cue valence, and cue arousal from mixed-effects models predicting each of the seven dependent variables. Retrieval success was a binary variable (higher values = greater likelihood of retrieving a memory), retrieval times were measured in seconds, and all other dependent variables were measured on 5-point rating scales. Error bars represent one standard error of the mean.

In the subsequent analyses, we tested the main effects and interactions of familiarity, emotional valence, and emotional arousal of the musical cues on the accessibility, emotional content, and other key qualities of the autobiographical memories. Descriptive statistics are reported in Table 1. Results from the significance tests for the mixed-effects models are reported in Table 2, and EMMs for all fixed factors from the mixed-effects models are displayed in Figure 1.

**Table 1.** Means and standard deviations by cue familiarity, valence, and arousal for all dependent variables.

Dependent measure	High-familiarity cues, *M* (*SD*)	Low-familiarity cues, *M* (*SD*)	Positive valence cues, *M* (*SD*)	Negative valence cues, *M* (*SD*)	High-arousal cues, *M* (*SD*)	Low-arousal cues, *M* (*SD*)
Number of memories	**7.67 (2.73)**	**2.80 (2.57)**	5.13 (2.36)	5.26 (2.31)	5.36 (2.26)	5.03 (2.41)
Retrieval time (s)	**10.46 (4.06)**	**13.65 (4.56)**	10.74 (4.42)	11.32 (3.93)	11.14 (4.38)	11.13 (4.33)
Memory valence rating	**3.73 (0.59)**	**3.48 (0.82)**	**3.90 (0.68)**	**3.43 (0.68)**	**3.81 (0.69)**	**3.49 (0.77)**
Memory arousal rating	**3.38 (0.68)**	**2.85 (0.90)**	**3.49 (0.65)**	**3.04 (0.71)**	**3.92 (0.74)**	**2.51 (0.70)**
Memory vividness rating	3.15 (0.82)	3.43 (0.98)	3.22 (0.83)	3.20 (0.92)	3.17 (0.83)	3.24 (0.91)
Memory uniqueness rating	2.90 (0.80)	2.98 (1.01)	2.80 (0.87)	2.98 (0.81)	2.95 (0.84)	2.92 (0.85)
Memory importance rating	2.53 (0.71)	2.39 (0.94)	2.60 (0.87)	2.46 (0.72)	**2.42 (0.80)**	**2.69 (0.86)**

Values in bold correspond to significant main effects found in the mixed-effects models (see Table 2).

**Table 2.** Results of Wald χ^2^ tests assessing the statistical significance of the fixed effects and interactions of cue familiarity, valence, and arousal on each dependent variable.

Dependent measure	Predictor	χ^2^	*p*
Retrieval success (pseudo-*R*^2^ = .31)	Familiarity	451.29	<.001*
	Valence	0.73	.39
	Arousal	0.17	.68
	Familiarity × Valence	0.25	.62
	Familiarity × Arousal	3.50	.06
	Valence × Arousal	0.01	.93
	Familiarity × Valence × Arousal	4.01	.05
Retrieval time (pseudo-*R*^2^ = .06)	Familiarity	59.08	<.001*
	Valence	2.41	.12
	Arousal	0.52	.47
	Familiarity × Valence	0.14	.71
	Familiarity × Arousal	0.26	.61
	Valence × Arousal	4.25	.04
	Familiarity × Valence × Arousal	1.37	.24
Memory valence rating (pseudo-*R*^2^ = 0.10)	Familiarity	11.17	.001*
	Valence	58.79	<.001*
	Arousal	19.95	<.001*
	Familiarity × Valence	1.10	.29
	Familiarity × Arousal	6.44	.01
	Valence × Arousal	9.87	.002*
	Familiarity × Valence × Arousal	1.27	.26
Memory arousal rating (pseudo-*R*^2^ = .34)	Familiarity	30.22	<.001*
	Valence	30.62	<.001*
	Arousal	411.45	<.001*
	Familiarity × Valence	1.16	.28
	Familiarity × Arousal	6.87	.01
	Valence × Arousal	4.04	.05
	Familiarity × Valence × Arousal	4.44	.04
Memory vividness rating (pseudo-*R*^2^ = .01)	Familiarity	6.57	.01
	Valence	0.01	.91
	Arousal	0.46	.50
	Familiarity × Valence	1.59	.21
	Familiarity × Arousal	0.01	.92
	Valence × Arousal	0.21	.65
	Familiarity × Valence × Arousal	0.35	.55
Memory uniqueness rating (pseudo-*R*^2^ = .01)	Familiarity	0.73	.39
	Valence	1.61	.20
	Arousal	0.46	.50
	Familiarity × Valence	0.25	.62
	Familiarity × Arousal	5.64	.02
	Valence × Arousal	1.53	.22
	Familiarity × Valence × Arousal	0.01	.94
Memory importance rating (pseudo-*R*^2^ = .03)	Familiarity	4.81	.03
	Valence	6.99	.008
	Arousal	7.81	.005*
	Familiarity × Valence	0.17	.68
	Familiarity × Arousal	2.71	.10
	Valence × Arousal	5.35	.02
	Familiarity × Valence × Arousal	1.22	.27

Retrieval success was predicted via a binomial mixed-effects model; all other dependent variables were predicted using linear mixed-effects models. “Participant” was included as a random effect in all models. Nagelkerke pseudo-*R*^2^ values were computed for each model by comparing the fitted model against a null (intercept-only) model.

* *p* < .0071 (Bonferroni-corrected for seven dependent variables).

#### Accessibility of memories

The binomial mixed-effects model predicting retrieval success revealed one significant effect, specifically, a main effect of familiarity, with high-familiarity stimuli significantly more likely to evoke a memory than low-familiarity stimuli (see Tables 1 and 2, and Figure 1). On average, across the participant sample, high-familiarity stimuli evoked 7.67 memories (*SD* = 2.73) and low-familiarity stimuli evoked 2.80 memories (*SD* = 2.57). In a linear mixed-effects model to predict memory retrieval times, a significant main effect of familiarity was also found, with high-familiarity cues eliciting memories more quickly (lower retrieval times) than low-familiarity cues.

#### Emotional content of memories

Analyses of the valence ratings for the memories revealed a main effect of familiarity, with high-familiarity music eliciting more positive memories than the low-familiarity music.

**Figure 2.**
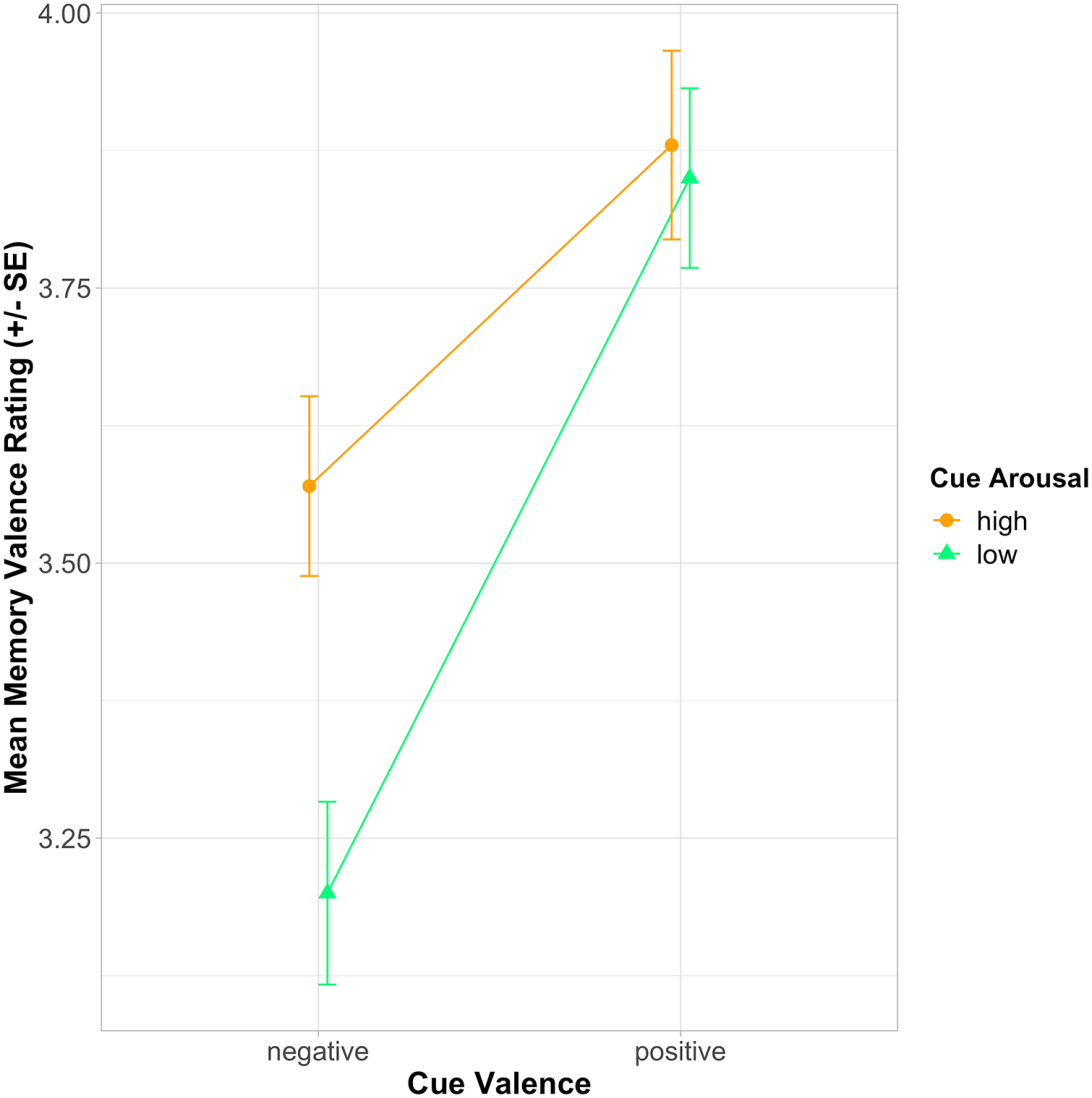
Estimated marginal means from linear mixed-effects model of memory valence ratings by cue valence and cue arousal. Error bars denote one standard error of the mean. Higher memory valence ratings indicate more positive memories.

Ratings of the valence of the retrieved memories were significantly predicted by the valence and arousal of the cues, with a significant interaction between valence and arousal (see Figure 2). Specifically, in post hoc *t*-tests with Bonferroni correction for multiple comparisons, positively valenced cues elicited relatively positive memories, regardless of whether they were high or low in arousal (*p* > .99). However, for the negatively valenced cues, those high in arousal elicited significantly more positive memories than those low in arousal (*p* = .002). The negative valence/low-arousal cues evoked significantly less positive memories than both positive cue types (*p*s < .001), and the negative valence/high-arousal cues also evoked significantly less positive memories than both positive cue types (*p*s < .05).

As memory valence was rated on a bipolar scale from “*very negative*” to “*very positive*” (rather than as an increasing quantity of a particular construct such as “vividness” or “energy”), we also examined the distribution of “negative” and “positive” memories by categorising memories as “*negative*” if they were rated 1 or 2 on the memory valence rating scale and “*positive*” if they were rated 4 or 5 on this scale. Ratings of 3 (the midpoint of the scale) were excluded from consideration here. The number and percentage of memories falling into each of these categories, as a function of cue valence and cue arousal are shown in Table 3. From Table 3, it is apparent that although negative memories were less common than positive memories, the negative valence/low-arousal cues elicited both more negative and fewer positive memories than the other cue types, mirroring the analysis reported above.

**Table 3.** Number (and percentage) of positive and negative autobiographical memories reported, by cue valence and cue arousal.

	Memory valence	
Cue type	Positive	Negative
Positive valence/high arousal	185 (30%)	20 (13%)
Positive valence/low arousal	165 (27%)	21 (13%)
Negative valence/high arousal	162 (26%)	37 (24%)
Negative valence/low arousal	110 (18%)	79 (50%)
Total	622 (100%)	157 (100%)

The valence and arousal of the cues also both showed significant main effects on ratings of memory arousal, with high-arousal cues eliciting more arousing memories and positively valenced cues also eliciting more arousing memories. In this case, arousal and valence did not significantly interact. In addition, high-familiarity stimuli evoked significantly more arousing memories than low-familiarity stimuli.

#### Memory qualities

Ratings of the importance of the memories were significantly affected by the arousal of the cues, with low-arousal cues evoking more important memories than high-arousal cues. No significant effects were found in the models predicting vividness or uniqueness ratings of the memories.

### Liking of musical cues

Previous research has shown that familiarity of musical stimuli is typically correlated with liking ratings of such stimuli (Jakubowski et al., 2020; Janata et al., 2007; Krumhansl & Zupnick, 2013). This was indeed the case in our study: stimuli in the high-familiarity music category were as significantly more liked (*M* = 3.79, *SD* = 0.58, 95% CI = [3.67, 3.90]) than those in the low-familiarity category (*M* = 2.51, *SD* = 0.53, 95% CI = [2.41, 2.62]), *t*(99) = 20.73, *p* < .001. For each participant, we calculated the correlation between familiarity and liking of the stimuli; the mean value of these correlations across participants was .57 (*SD* = .20, range = .09–.93).

To understand whether the familiarity-related effects reported above might actually be driven by liking of the stimuli, we reran all analyses in which familiarity had a significant effect on memory properties (i.e., retrieval success, retrieval times, memory valence ratings, memory arousal ratings) with cue liking ratings included as a covariate. These results are presented in Supplementary Table 1. In the model predicting retrieval success, liking ratings were a significant positive predictor of whether a memory was retrieved (β = 0.46, *SE* = 0.05), but familiarity of the stimuli also still made a significant, independent contribution to the model, with high-familiarity stimuli (EMM = 0.70, *SE* = 0.13) more likely to evoke a memory than low-familiarity stimuli (EMM = –1.14, *SE* = 0.13). For retrieval times, higher liking ratings predicted significantly shorter (i.e., faster) retrieval times (β = –0.53, *SE* = 0.19) but cue familiarity was still a significant predictor, with high-familiarity cues (EMM = 10.5, *SE* = 0.38) requiring shorter retrieval times than low-familiarity cues (EMM = 13.2, *SE* = 0.50). For memory valence ratings, higher liking ratings predicted significantly more positive memories (β = 0.47, *SE* = 0.03), and the previously seen effect of cue familiarity was now reversed from the initial model presented in Table 1. Specifically, when liking was accounted for, high-familiarity music (EMM = 3.63, *SE* = 0.05) now evoked less positive memories than low-familiarity music (EMM = 3.85, *SE* = 0.07). Finally, for memory-arousal ratings, higher liking ratings predicted significantly more arousing memories (β = 0.32, *SE* = 0.03), but cue familiarity was no longer a statistically significant predictor in the model.

## Discussion

We tested effects of the familiarity, emotional valence, and emotional arousal of musical cues on properties of autobiographical memory recall. The familiarity and emotional expression of the cues were both found to influence aspects of the memories, with a somewhat different pattern of results emerging for each of these predictors.

### Familiarity of cues primarily affects accessibility of memories

The accessibility of autobiographical memories was primarily affected by the familiarity of the cues. Our high-familiarity musical stimuli evoked around 3 times as many memories as the low-familiarity stimuli, which extends previous findings that musical familiarity is correlated with autobiographical recall (Jakubowski et al., 2020; Janata et al., 2007; Krumhansl & Zupnick, 2013; Schulkind et al., 1999). This is also in line with research showing that more familiar visual cues evoke more autobiographical memories (Robin et al., 2019; Robin & Moscovitch, 2014, 2017), indicating that music operates similarly to other retrieval cues on this dimension.

We also found, in follow-up analyses, that cue familiarity and cue liking were both independent positive predictors of whether a memory was evoked by a cue. Thus, even when liking is accounted for, high-familiarity music is more likely to evoke a memory than low-familiarity music. This may be because highly familiar music is likely to be associated with a range of autobiographical events even if the music is not personally valued. For instance, chart-topping songs such as those used here are frequently encountered in everyday situations in which the listener does not necessarily choose a particular song (e.g., shops, dances, parties, clubs, TV/films) (Krause et al., 2015; Sloboda et al., 2001), and therefore may become incidentally associated with memories of life events regardless of whether the listener likes the music.

The high-familiarity music also evoked memories significantly more quickly, suggesting a more direct retrieval process. A similar effect of cue familiarity on memory-retrieval times has been found in previous research using visual cues (Robin & Moscovitch, 2014). In our study, on approximately 79% of high-familiarity music trials in which a memory was retrieved, participants reported that the piece of music used as a cue was present at encoding of the event. This indicates that the high-familiarity music provided a closer cue-target match to an autobiographical memory than the low-familiarity music. Subsequent analysis revealed that this main effect of cue familiarity on retrieval times was maintained even when liking ratings were included in the model, while greater cue liking also independently predicted significantly faster retrieval times. One potential explanation for this relationship between liking ratings and retrieval times is that liking ratings may have served as an additional, complementary index of previous exposure to particular songs or musical styles. For instance, for the high-familiarity music, it is likely that participants had previously heard high-familiarity songs that they liked more often over their lifetimes, or more recently, than high-familiarity songs they did not particularly like. For most trials in which a memory was recalled in response to low-familiarity music (58%), participants reported they had never heard the song before. Nevertheless, in this case, liking ratings could be an index of general musical tastes; for instance, a high liking rating given to a piece of rock music might indicate that a particular participant is a fan of rock music and had heard many similar-sounding songs before. Such cues might thereby elicit faster retrieval of memories as participants could draw upon many (and perhaps more recent) memories of listening to similar songs, whereas it may require significantly more time to recall a memory related to a style of music that is not to one’s personal taste. Future research should consider the musical tastes of each participant, to further understand how the range of genres used here might impact properties of the retrieved memories depending on personal preferences and exposure patterns to particular genres or styles.

High-familiarity cues also evoked significantly more positive memories and significantly more arousing memories than low-familiarity cues. This replicates findings from Ford et al. (2016) on cue valence, and extends these findings to cue arousal. However, our follow-up analyses provide further insight into this result by showing that these differences in the valence and arousal of memories evoked by high- versus low-familiarity music may actually be attributed to differences in liking between these two sets of cues. Specifically, cue familiarity no longer had a significant effect on memory arousal and the relationship between cue familiarity and memory valence was reversed^7^ when cue liking was added to the model, while cue liking was a significant positive predictor of both memory valence ratings and memory arousal ratings. One potential explanation for these results is therefore that, because it was more liked, the more familiar music may have induced a more positive and energetic mood, resulting in more positive, more arousing memories being retrieved. This aligns with previous findings that other types of mood manipulations stimulate the recall of affectively congruent memories (Blaney, 1986; Bower, 1981; Singer & Salovey, 1988).

### Emotional expression of cues primarily affects emotional content and evaluation of memories

The emotional expression of the musical cues impacted the emotional content of the memories they evoked. Both positive valence and high-arousal cues led to more positive memories, with a significant interaction between these two predictors. While, as anticipated, both positive cue types (regardless of arousal level) elicited relatively positive memories (see Figure 2), we also found that the negative valence/high-arousal cues elicited more positive memories than the negative valence/low-arousal cues (see Schulkind & Woldorf, 2005, for a similar result). This may potentially be explained by the situational contexts and functions for which such music is typically employed, at least in the Western society in which this experiment was run (Olsen et al., 2022; Taruffi & Koelsch, 2014). For instance, upbeat, “angry” sounding music (negative valence/high arousal) is often played in social settings, such as clubs or parties, where people gather and dance with friends, which could thereby link such music to positive memories. Indeed, memory descriptions associated with music falling into this emotion category often mentioned instances of dancing, singing, and listening to music with friends, such as “Dancing in a club with friends on a Cheesy Classics night” and “Being in the crowd with a big group of friends at a music festival.” Conversely, “sad” sounding music (negative valence/low arousal) may be more likely to be utilised during negative life events, such as funerals and breakups, and in solitary listening settings (Taruffi & Koelsch, 2014); examples from the current dataset include “I was at my nan’s funeral and music similar to this was played” and “I listened to this song at my friend’s house when I was 14 when I had my first childhood heartbreak.”

In addition, as anticipated, high-arousal cues evoked more arousing memories. Cue valence also significantly impacted memory arousal (to a lesser extent than cue arousal), such that positive cues elicited more arousing memories. Taken together, these results indicate that both the valence and arousal of musical cues impact both the valence and arousal of autobiographical memories, but that these two emotional dimensions are not entirely dissociable.

Previous studies on the effects of the emotional expression of musical cues on autobiographical memories have used solely unfamiliar music (Jakubowski & Eerola, 2022; Schulkind & Woldorf, 2005; Sheldon & Donahue, 2017; Sheldon et al., 2020). The results of our experiment indicate that the emotional expression of familiar music exhibits highly similar effects on autobiographical memories to those previously found for unfamiliar music. Given that, in most instances, our participants reported that the high-familiarity cues were present at encoding of the original event, this suggests that music typically accompanies autobiographical events in an emotionally congruent manner. That is, positive music tends to be played during positive life events and vice versa, while more energetic music tends to accompany more energetic events. A primary exception here is the example of negative valence/high-arousal (e.g., “angry”) music, which seems to be coupled with more positive events than would be expected; in this case, it appears the upbeat (arousing) nature of the music serves to at least partially counteract its negative expression. Alternatively, it may be that events that were originally experienced in conjunction with emotionally congruent music were better encoded, and thus more readily recalled, than life events that were accompanied by non-emotionally congruent music (cf., Tesoriero & Rickard, 2012). A final potential explanation for these findings is that the valence and arousal of the musical cues influenced participants’ judgements of the emotionality of the events at recall; for instance, positive music cues caused events to be judged as more positive than perhaps they originally were, or more positive than they might be judged if recalled in response to a more emotionally neutral cue. Indeed, it is possible that the present results may be attributed to some combination of these explanations, and future research that also captures emotional responses to events at encoding is needed to further tease apart these contributing factors.

The extent to which autobiographical memories were rated as personally important was also related to the emotional expression (but, interestingly, not familiarity) of the musical cues. Specifically, low-arousal cues elicited recall of more important memories. This same pattern of results was reported in a recent study using word cues to evoke autobiographical memories (Simpson & Sheldon, 2020), suggesting that the emotional expression of music impacts the recall of personally valued memories in an analogous way to other retrieval cues.

The methodology developed here could be utilised in future research to test whether our findings on the influence of the emotional expression of a cue on autobiographical memory properties extend to other, non-musical cues. Previous studies have compared the features of autobiographical memories evoked via emotional versus neutral word cues (Maki et al., 2013; Robinson, 1976), for instance, but a more nuanced approach in which retrieval cues are classified according to both their valence and arousal, or according to discrete emotion categories, could shed further light on how cue emotionality affects autobiographical memory. One recent study along these lines used two emotion words from each of the four valence/arousal categories (e.g., “thrilled,” “proud,” “panic,” “hopeless”) as autobiographical memory cues (Simpson & Sheldon, 2020). Given that they did not find the same divergences we did between negative valence/high-arousal and negative valence/low-arousal cues in ratings of memory valence (see Figure 2), this suggests such effects are specific to the sociocultural contexts in which music is used in everyday life.^8^ However, future studies using a wider range of cue types, and specifically those comparing music to other aesthetic objects (e.g., artworks), could further elucidate whether music might be different to other retrieval cues in this regard.

### Additional results and future directions

The lack of interactions between familiarity and emotional expression indicate that these two properties of musical cues operate relatively independently in their effects on autobiographical memories. This indicates, for example, that the emotional expression of musical cues has similar effects on the emotional content and evaluation of memories regardless of how familiar the music is to the listener. Thus, research aiming to investigate the impact of musical cues on the emotional content of memories should be able to utilise either familiar or unfamiliar music and expect similar effects in this regard.

The models predicting ratings of vividness and uniqueness of the memories did not reveal any significant effects. It is somewhat surprising that cue familiarity did not impact vividness ratings, given some previous evidence of a relationship between these factors in the autobiographical memory literature (Robin et al., 2019; Robin & Moscovitch, 2014, 2017). Future research in this area could solicit more detailed memory descriptions from participants to explore whether a qualitative coding approach (such as that used in the Autobiographical Interview; Levine et al., 2002) reveals any further differences in terms of the quantity and type of episodic details evoked. This may provide a more nuanced insight than the 5-point memory vividness rating scale used here.

In addition, some previous research has suggested that more familiar music elicits more specific memories, particularly in young adults (Ford et al., 2016). However, we did not find a relationship between cue familiarity and a similar measure to specificity—ratings of the uniqueness of the memories. This may be due to a methodological difference. In our study, we followed the method used by Sheldon and Donahue (2017), asking participants to retrieve a memory of a specific event (lasting no longer than one day) and rate how unique it was (on a scale from “1 = *not at all—this type of event happens all the time*” to “5 = *extremely unique—once in a lifetime event*”). Ford et al. (2016) captured a wider range of levels of memory specificity, including those of extended (e.g., week-long) events. Thus, future research that includes memories of more extended events may reveal findings more in line with Ford et al. (2016).

To maximise the familiarity/unfamiliarity of the musical cues, we tested a relatively homogeneous sample of participants (young adults in the United Kingdom). Future research should extend our approach to other age groups; for instance, it is unknown whether the familiarity effects found here might be stronger in older age groups due to the increased exposure to familiar music that may have accrued over their longer lifetimes. Our results are also likely constrained by cultural usages of music within the United Kingdom (and perhaps similar western cultures). For example, although it is common to play sombre music at funerals in this society, in various other cultures, funerals may be accompanied by more joyful, upbeat music with the aim of celebrating the life (and/or afterlife) of the deceased (Krueger, 2019). Thus, the associations between emotional music and emotional events are likely mediated by culture in many ways that have been highly under-investigated to date.

## Conclusion

In conclusion, we have revealed that the familiarity and emotional expression of musical cues affect different features of autobiographical memories. Specifically, more familiar music elicited more memories and invoked shorter memory retrieval times. More familiar music also evoked more positive and arousing memories, although these effects were found to be attributed to the fact that more familiar music was also more liked. The valence and arousal of the musical cues affected the emotional tone and importance ratings of the memories.

Almost all of the effects found here have parallels in autobiographical memory research using pictorial or word cues (Robin et al., 2019; Robin & Moscovitch, 2014; Simpson & Sheldon, 2020), suggesting that music affects autobiographical recall in a highly similar way to other common retrieval cues. This offers strong counterevidence to the common notion that music is “special” as a cue for autobiographical memories (see also Halpern et al., 2018). One notable exception is the divergence we found between negative valence/high-arousal and negative valence/low-arousal cues, in which the former evoked more positive memories than the latter; no such interaction between cue valence and cue arousal was found in a similar study using word cues (Simpson & Sheldon, 2020). This suggests that the everyday contexts in which emotional music is employed may elicit some differential effects on memory properties in comparison to other retrieval cues.

Our results indicate that features of the stimuli should be taken into account when employing music as an autobiographical memory cue and, crucially, when comparing it against other cues. In particular, controlling for features including familiarity, emotional expression, and liking will help to prevent spurious conclusions being made about the “power of music” to evoke autobiographical memories that may actually be driven by differences in features of musical versus non-musical cues. More broadly, this work furthers our understanding of the mapping between particular musical cues and autobiographical memories, in terms of how aspects of these retrieval cues direct and constrain memory recall processes. Research in this domain has the potential to inform uses of music in a variety of applied contexts, such as therapy and advertising, in which it may be desired to evoke autobiographical memories with specific qualities, such as particularly positive or personally valued memories.

## Supplemental Material

sj-docx-1-qjp-10.1177_17470218221129793 – Supplemental material for Differential effects of familiarity and emotional expression of musical cues on autobiographical memory properties Click here for additional data file. Supplemental material, sj-docx-1-qjp-10.1177_17470218221129793 for Differential effects of familiarity and emotional expression of musical cues on autobiographical memory properties by Kelly Jakubowski and Emma Francini in Quarterly Journal of Experimental Psychology

## Notes

1 One exception here is that several of these studies matched musical and non-musical cues in terms of their date of likely exposure (e.g., comparing famous faces and music that were popular during the same time period; Belfi et al., 2016).

2 Looping was required for 10 high-familiarity stimuli and 2 low-familiarity stimuli to meet the desired stimulus duration of 30 s. It should be noted, however, that even stimuli that were not looped often contained many repetitions of the same musical material; for instance, the low-familiarity stimulus “346” contained two iterations of the same sections, with each section comprising two to four iterations of the same motive. This is in line with the generally highly repetitive nature of music, as noted in various previous research (see Margulis, 2013, for an overview).

3 https://www.officialcharts.com/chart-news/the-best-selling-singles-of-all-time-on-the-official-uk-chart__21298/.

4 *Positive valence* = mean valence rating greater than 5; *Negative valence* = mean valence rating less than 5; *High arousal* = mean arousal rating greater than 5; *Low arousal* = mean arousal rating less than 5

5 Self-reported “yes” responses to the familiarity question were also highly correlated (*r*s > .85) with our measures of whether participants could correctly name the title and artist of the song.

6 This is likely an underestimation of true familiarity, as it is a common experience to recognise a song without knowing its exact title. We also adopted a relatively strict scoring protocol for these data. Instances in which a word was added or excluded from the title, such as “Don’t Wake Me Up” as a response to the song “Wake Me Up,” were marked as incorrect. However, we allowed for the exclusion of articles, for example, “Final Countdown” was accepted as a correct answer for the song “The Final Countdown.”

7 High-familiarity cues evoked less positive memories than low-familiarity cues.

8 Specifically, in their study cue valence affected memory valence ratings (more positive cues evoked more positive memories), but cue valence did not interact with cue arousal.
