# Supplementary material for: Differential effects of familiarity and emotional expression of musical cues on autobiographical memory properties
Source: Q J Exp Psychol (Hove). 2022 Oct 27;76(9):2001–16. doi: 10.1177/17470218221129793 (PMC10466948; doi:10.1177/17470218221129793)
Supplement: sj-docx-1-qjp-10.1177_17470218221129793 – Supplemental material for Differential effects of familiarity and emotional expression of musical cues on autobiographical memory properties [file sj-docx-1-qjp-10.1177_17470218221129793.docx]

Supplementary Material for:

**Differential Effects of Familiarity and Emotional Expression of Musical Cues on Autobiographical Memory Properties**

Kelly Jakubowski^1^ & Emma Francini^2^

^1^Department of Music, Durham University, UK

^2^ Department of Psychology, Durham University, UK

**Supplementary Material A: Main Experiment Questions**

Demographic questions (response options in italics)

Age (in years)

*Dropdown menu ranging from 18 to 100*

Gender

*Male; Female; Other; Prefer not to say*

In which country do you currently live?

*Dropdown menu*

What is your first (native) language?

*Open response*

Do you currently suffer from any hearing impairments?

*Yes; No*

[If “yes” response to above] Please provide a short description of the hearing impairment, and any measures you are currently taking to correct it (wearing a hearing aid, etc.).

*Open response*

Memory feature questions (response options in italics)

How vivid is the memory in your mind? In other words, how clear is the image of the event in your mind?

*1 (not at all vivid); 2; 3; 4; 5 (extremely vivid)*

Please rate the emotional content of the memory, in terms of how negative or positive it is.

*1 (very negative); 2; 3; 4; 5 (very positive)*

How energetic is the content of this memory?

*1 (not at all energetic); 2; 3;4; 5 (extremely energetic)*

Please provide a short (1 sentence) description of the memory that you recalled, including details of what you were doing, who you were with, and where you were in the remembered event. (Note: If this is a memory, you do not wish to share the content of for personal reasons, please simply write 'Private'.)

*Open response*

How old were you (in years) during the event that you recalled? If unsure, please give your best estimate.

*Dropdown menu ranging from 18 to 100*

How unique is the event that you remembered?

*1 (not at all- this type of event happens all the time); 2; 3; 4; 5 (extremely unique- once in a lifetime event)*

How important is this memory to your life story?

*1 (not at all important); 2; 3; 4; 5 (extremely important)*

Was the piece of music that you just heard present during the event that you recalled? That is, did your memory involve a previous incident of listening to this same piece of music?

*Yes; No; Not sure*

Musical stimuli questions (response options in italics)

How much did you like the piece of music you just heard?

*1 (dislike a lot); 2; 3; 4; 5 (like a lot)*

How familiar are you with the piece of music you just heard?

*1 (never heard it before); 2; 3; 4; 5 (have frequently heard it)*

[If familiarity rating of 2 or greater above] Please type the name of the song, to the best of your memory, here. If unsure please make a guess.

*Open response*

Musicianship questions (response options in italics)

Which of the following best describes you?

*Non-musician; music-loving non-musician; amateur musician; serious amateur musician; semi-professional musician; professional musician*

I have had __ years of formal training on a musical instrument (including voice) during my lifetime.

*0; 0.5; 1; 2; 3-5; 6-9; 10 or more*

**Supplementary Material B: Musical Stimuli**

| Stimulus number | Familiarity category | Emotion category | Song title | Artist | Genre | Release year |
| --- | --- | --- | --- | --- | --- | --- |
| 1 | High | Positive valence/Low arousal | Careless Whisper | George Michael | Pop | 1984 |
| 2 | High | Positive valence/Low arousal | Here Comes the Sun | The Beatles | Classic rock | 1969 |
| 3_high* | High | Positive valence/Low arousal | Thinking out Loud | Ed Sheeran | Soul | 2014 |
| 4 | High | Positive valence/High arousal | Moves Like Jagger | Maroon 5 ft Christina Aguilera | Dance/Pop | 2011 |
| 5 | High | Positive valence/High arousal | Wake me up | Avicii | Electronic | 2013 |
| 6 | High | Positive valence/High arousal | Walking on Sunshine | Katrina & The Waves | Pop-rock/New wave | 1985 |
| 7 | High | Negative valence/Low arousal | Someone Like You | Adele | Pop/Soul | 2011 |
| 8 | High | Negative valence/Low arousal | Let her go | Passenger | Folk | 2012 |
| 9 | High | Negative valence/Low arousal | 7 Years | Lukas Graham | Soul-pop | 2015 |
| 10 | High | Negative valence/High arousal | The Final Countdown | Europe | Hard rock | 1986 |
| 11 | High | Negative valence/High arousal | Toxic | Britney Spears | Dance/Pop | 2014 |
| 12 | High | Negative valence/High arousal | Smooth Criminal | Michael Jackson | Pop | 1988 |
| 1116** | Low | Positive valence/Low arousal | If | Peter Rudenko | Classical | NA |
| 1530 | Low | Positive valence/Low arousal | Want You | Deal the Villain | Hip-hop | NA |
| 1749 | Low | Positive valence/Low arousal | Empingue | Gepel | Jazz | NA |
| 346 | Low | Positive valence/High arousal | Tennesee Hayride | Jason Shaw | Country | NA |
| 1828 | Low | Positive valence/High arousal | Natalie | The Sand Pebbles | Pop | NA |
| 1913 | Low | Positive valence/High arousal | Paranoia | The Bomb Busters | Rock | NA |
| 3 | Low | Negative valence/Low arousal | DD Groove | Kevin MacLeod | Blues | NA |
| 190 | Low | Negative valence/Low arousal | Virtutes Instrumenti | Kevin MacLeod | Classical | NA |
| 1469 | Low | Negative valence/Low arousal | Siren | The Family Simpson | Folk | NA |
| 1334 | Low | Negative valence/High arousal | Eyes | I Have Clones | Electronic | NA |
| 1788 | Low | Negative valence/High arousal | Set the Fire | Jooklo Duo with C. Spencer Yeh | Experimental jazz | NA |
| 1948 | Low | Negative valence/High arousal | Joan | The Asound | Rock | NA |

*“high” label added due to the fact that we also used stimulus 3 from the DEAM dataset (low familiarity category);

** numberings of the low familiarity stimuli are identical to those used in the DEAM dataset;

Genre classifications for high familiarity stimuli were sourced from Last.fm, and cross-checked with Wikipedia entries for each song. Genre classifications for low familiarity stimuli are taken from the DEAM metadata.


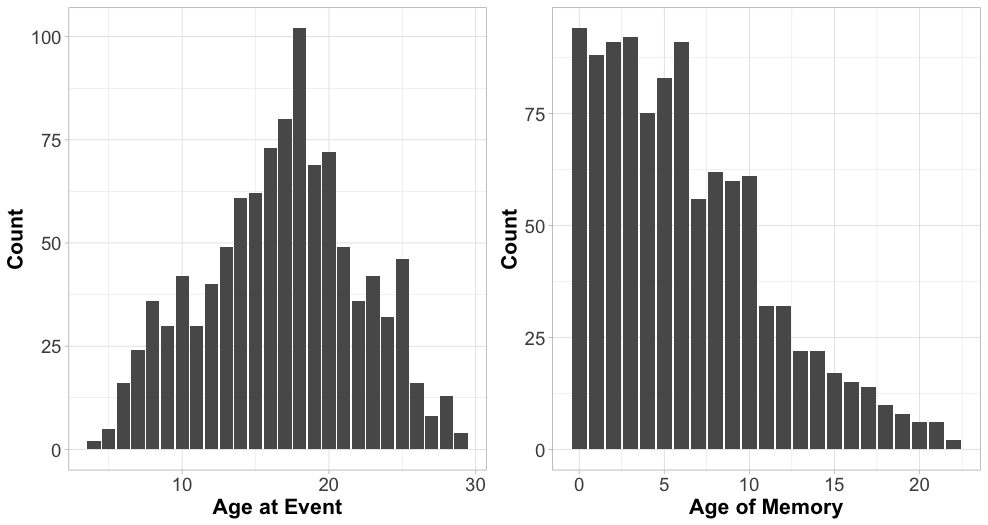


Supplementary Figure 1. All autobiographical memories used in the analyses (*N* = 1,039) by participant’s age during the remembered event (left panel) and the age of the memory (i.e., participant’s current age minus age at event; right panel).

Supplementary Table 1. Results of Wald χ^2^ tests assessing the statistical significance of the fixed effects and interactions of cue familiarity, valence, and arousal on retrieval success, retrieval time, and memory valence ratings, and memory arousal ratings, with cue liking ratings included as a covariate. Retrieval success was predicted via a binomial mixed effects model; all other dependent variables were predicted using linear mixed effects models. “Participant” was included as a random effect in all models. Nagelkerke pseudo-*R^2^* values were computed for each model by comparing the fitted model against a null (intercept-only) model.

| Dependent measure | Predictor | *χ^2^* | *p* |
| --- | --- | --- | --- |
| Retrieval success  (Pseudo-*R*^2^ = 0.34) | Familiarity | 220.40 | < .001* |
|  | Valence | 3.18 | .07 |
|  | Arousal | 4.19 | .04 |
|  | Liking | 74.24 | < .001* |
|  | Familiarity × Valence | 0.10 | .75 |
|  | Familiarity × Arousal | 0.27 | .60 |
|  | Valence × Arousal | 0.04 | .84 |
|  | Familiarity × Valence × Arousal | 6.33 | .01 |
| Retrieval time  (Pseudo-*R*^2^ = 0.07) | Familiarity | 34.64 | < .001* |
|  | Valence | 1.96 | .16 |
|  | Arousal | 0.13 | .72 |
|  | Liking | 7.57 | .006* |
|  | Familiarity × Valence | 0.31 | .58 |
|  | Familiarity × Arousal | 0.02 | .88 |
|  | Valence × Arousal | 4.36 | .04 |
|  | Familiarity × Valence × Arousal | 1.74 | .19 |
| Memory valence rating  (Pseudo-*R*^2^ = 0.28) | Familiarity | 8.48 | .004* |
|  | Valence | 56.39 | < .001* |
|  | Arousal | 48.15 | < .001* |
|  | Liking | 231.06 | < .001* |
|  | Familiarity × Valence | 4.51 | .03 |
|  | Familiarity × Arousal | 0.67 | .41 |
|  | Valence × Arousal | 11.88 | <.001* |
|  | Familiarity × Valence × Arousal | 0.15 | .70 |
| Memory arousal rating  (Pseudo-*R*^2^ = 0.39) | Familiarity | 1.77 | .18 |
|  | Valence | 26.77 | < .001* |
|  | Arousal | 491.32 | < .001* |
|  | Liking | 90.88 | < .001* |
|  | Familiarity × Valence | 2.98 | .08 |
|  | Familiarity × Arousal | 2.32 | .13 |
|  | Valence × Arousal | 4.25 | .04 |
|  | Familiarity × Valence × Arousal | 7.27 | .007* |

Note: * = *p* < .0071 (Bonferroni corrected for 7 dependent variables, as in the original analysis; see Table 2 in main article)
